# A Novel Small RNA Regulates Tolerance and Virulence in *Shigella flexneri* by Responding to Acidic Environmental Changes

**DOI:** 10.3389/fcimb.2016.00024

**Published:** 2016-03-08

**Authors:** Ligui Wang, Guang Yang, Lihua Qi, Xiang Li, Leili Jia, Jing Xie, Shaofu Qiu, Peng Li, RongZhang Hao, Zhihao Wu, Xinying Du, Wuju Li, Hongbin Song

**Affiliations:** ^1^Institute of Disease Control and Prevention, Academy of Military Medical SciencesBeijing, China; ^2^Center of Computational Biology, Beijing Institute of Basic Medical SciencesBeijing, China

**Keywords:** sRNA, *Shigella flexneri*, response, stress tolerance, virulence

## Abstract

*Shigella flexneri* is an important cause of bacillary dysentery in developing countries. Small regulatory RNAs (sRNAs) play essential roles in diverse cellular processes. We found a novel sRNA *Ssr1* based on RT-PCR, northern blot, and 5′RACE in *S. flexneri*. *Ssr1* responds to acidic environmental changes, as shown by a strong linear correlation between the pH value and *Ssr1* expression (*R* = 0.785, *P* < 0.05) using the qRT-PCR method. Deletion of *Ssr1* results in growth retardation at pH values ranging from 5.0 to 7.0 (*P* < 0.05), and the survival rate was reduced by 22% in acidic conditions (pH 3.0). Additionally, virulence was significantly increased in an *Ssr1* mutant strain, as revealed in a murine lung invasion model and survival model assays. By using the sTarPicker method and proteomic analysis, we considered that DnaK, which is a major factor that confers acidic stress tolerance, may be a direct target of *Ssr1*. We also found that *Ssr1* may enhance virulence by directly targeting OmpA; this leads to altered expression of genes in the type three secretion system (T3SS). This work provides new insight into the mechanism of adaptation to environmental stress and into the pathogenesis of *Shigella*.

## Introduction

Over the last few years, small regulatory RNAs (sRNAs)-based control mechanisms have been recognized as key regulators of gene expression. sRNAs vary from 50 to 500 nt in length and are characterized by base pairing with target mRNAs; this affects the activity of the target mRNA or its translated protein product. sRNAs are generally un-translated and regulate diverse physiological processes in bacteria, such as stress responses, metabolism, and virulence, as well as the control of the bacterial envelope's composition (Storz et al., 2006; Toledo-Arana et al., 2007; Vogel, 2009). Surprisingly, only a few sRNAs have roles in both responding to environmental stresses and regulating virulence (Gripenland et al., 2010). For instance, an sRNA (ef0408-0409) mutant strain of *Enterococcus faecalis* was able to grow and survive more effectively than wild-type bacteria in the presence of osmotic and oxidative stress, and it was more resistant to acidic stress. Furthermore, the strain was more virulent (Michaux et al., 2014). In *Shigella*, RyhB sRNA is involved in the response to the environmental iron level, and it regulates the expression of the type three secretion system (T3SS), which is the major source of virulence (Murphy and Payne, 2007; Marteyn et al., 2012).

Like other gram-negative enteric bacteria, *Shigella* encounter a gastric acidic pH (pH 2–3) before reaching the colon after oral infection (Cheng et al., 2007). *Shigella* have the ability to survive in extreme environmental conditions and are pathogenic in host cells during the infection process. Thus, *Shigella* express a set of transcriptional regulators to adapt to and survive in different enteric environmental conditions such as low pH, high temperature, and changing osmotic pressure (Murphy and Payne, 2007). Therefore, these bacteria have developed complex regulatory systems to respond to environmental signals (Papenfort and Vogel, 2014). Acidic stress adaptation confers resistance to a wide range of other stress conditions, including salinity, heat, and H_2_O_2_ (Cheng et al., 2007). This implies that the resistance to the gastrointestinal acidic environment is essential for pathogenesis in *Shigella*. Additionally, sRNAs are increasingly recognized as essential factors in the resistance to an acidic environment. As for an example, the GadY sRNA in *Escherichia coli* increases the expression of downstream acid resistance genes and regulates the GadX protein to increase the bacterial survival rate under low pH conditions (Opdyke et al., 2011).

Although a large number of *Shigella* strains have been sequenced, very little is known about sRNA identification and functions in this bacterium. Only nine sRNAs, *ffs, dsrA, micF, csrB, gcvB, ssrS, rnpS, spf*, and *oxyS*, have been annotated in the *Shigella* genome using comparative genomic methods. In a recent study, nine novel sRNAs were identified and validated by computer-based methods and northern blot analyses, but the characteristics of these sRNAs remain to be elucidated (Peng et al., 2011). Only two sRNAs, RnaG, and RyhB, have been studied comprehensively in *S. flexneri* (Peng et al., 2011). The production of sRNAs that control bacterial virulence is required to fine-tune signaling and survive diverse environmental conditions. In particular, there have been few reports regarding the role of sRNAs in the response to environmental signals that increase tolerance to extreme host environments, and in the regulation of virulence. In this study, we identified a novel sRNA, *Ssr1*, during a comparative genomics bioinformatic screen and experimentally verified that it regulates virulence and the tolerance response to environmental acidity.

## Materials and methods

### Bacterial strains and growth media

The strains used in this study are derivatives of the *S. flexneri* 2a 301 strain (the wild-type). *S. flexneri* strains were routinely cultured in LB medium (1% tryptone, 0.5% yeast extract and 1% NaCl) or on tryptic soy agar containing 0.01% (wt/vol) Congo red at 37°C. When required, ampicillin, kanamycin, and chloromycetin were added to final concentrations of 100, 50, and 30 μg/mL, respectively.

### Construction of *S. flexneri* 301 sRNA1 deletion strain and the complementation strain

The λ-Red-mediated recombination method was used to construct a *S. flexneri* 301 *Ssr1* deletion mutant (Δ*Ssr1*) by replacing the *Ssr1* gene with kanamycin resistance gene, encoding kanamycin resistance. Briefly, PCR was used to amplify regions of sequence upstream and downstream of the *Ssr1* gene using primer pairs (Park et al., 2001). Two ~500 bp sequences that overlapped the kanamycin resistance gene were fused by PCR with a complementary kanamycin resistance gene PCR fragment, resulting in the replacement of *Ssr1* with kanamycin resistance gene. The resulting PCR product was gel purified using a gel extraction kit (Promega, A9285). The fused PCR fragment was transformed into the wild-type. Δ*Ssr1* were identified by screening transformants on LB agar plates containing kanamycin (50 μg/mL).

To construct the complementing plasmid p*Ssr1*, the *Ssr1* coding region and 200 bp upstream of the transcriptional start site were amplified from the wild-type. These primers were designed to include unique restriction enzyme sites, *Xba*I and *Sph*I, so that when the PCR amplicon was digested with *Xba*I and *Sph*I, it could be ligated into a similarly digested plasmid, PACYC184, in an orientation-specific manner. Recombinant DNA products were verified by sequencing. The resulting plasmid was used to transform Δ*Ssr1* by electroporation, selecting for chloromycetin. The final complemented strain was called *p*sRNA1.

### sRNA prediction

Given that sRNAs are mainly located in the intergenic regions of the genome, we predicted the promoter and terminator in the intergenic regions of *Shigella flexneri*. The profile search program pftools2.3 (Lesnik et al., 2001) was used for promoter prediction, and RNAMotif (Berg and von Hippel, 1987; Livny et al., 2005) was used for rho-independent terminator prediction. Only the intergenic regions with simultaneously predicted promoter and rho-independent terminator were chosen for experimental verification.

### RNA isolation

Cells were harvested from *S. flexneri* cultured at 37°C during mid-log phase (OD_600_ of 0.4–0.6) by centrifugation. Total RNA from *S. flexneri* was prepared using the Trizol (Invitrogen, 15596108) procedure according to the manufacturer's instructions. RNA pellets were dissolved in DEPC-H_2_O. Total nucleic acid concentrations and purity were estimated using absorbance readings (260 nm/280 nm) on an Ultraspec II spectrophotometer.

### RT-PCR

The total RNA was treated with DNase according to the manufacturer's instructions (Promega, M6101). No more than 10 μg of total RNA was used to generate cDNA using a reverse transcription kit (Promega, K1005S) according to the product's directions. Each cDNA sample was diluted 1:10 in water, and 3 μL was used as the template for each 25-μL PCR. All probes were designed using Primer 5.0 (Listed as Table S1).

### Northern blotting

Northern blot analyses were carried out to confirm the transcription of sRNAs. A total of 18 candidate sRNAs were tested by northern blotting. Total RNA (15–20 μg) was separated by electrophoresis in a 10% polyacrylamide gel and transferred to a nylon membrane by electroblotting. RNAs were cross-linked to the membrane by exposure to ultraviolet light (Thermo Scientific, 89880). The membranes were hybridized with gene-specific biotin-labeled oligonucleotides, and hybridization signals were visualized using a Phosphor Imager (Molecular Dynamics).

### RACE

RACE experiments were performed according to the manufacturer's instructions (Takara Biochemicals, D315) to identify the 5′ ends of the cDNAs of interest. This method allows the discrimination of 5′ ends generated by transcription start sites and end sites.

### Stress tolerance assays

For growth experiments, overnight cultures grown in LB medium or LB medium containing kanamycin were diluted 1:100 in LB with the appropriate supplement and grown at 37°C with shaking at 160 rpm/min. For stress tolerance assays, the medium was adjusted to a specific pH level, 5.0, 6.0, 7.0, or 8.0. Bacterial growth was monitored by measuring the OD_600_.

For stress tolerance assays, the wild-type and Δ*Ssr1* were exposed to *in vitro* environmental stress conditions. Bacteria were inoculated into LB medium and grown to the early logarithmic phase (OD_600_ of 0.6–0.8) at 37°C. To determine the response to acidic stress, each strain was incubated at 37°C for 30 min in LB medium at pH 3.0. After the treatment, cells were diluted and plated on LB to determine the number of CFUs. Results represent the mean of at least three separate experiments.

### Sereny test

The sereny keratoconjunctivitis test was performed as described previously (Sereny, 1955) to evaluate the virulence of the wild-type and Δ*Ssr1*. Overnight bacterial cultures were serially diluted to suitable CFU/mL in NaCl for infection. A 20-μL drop of a 10^8^ CFU concentration was injected into the conjunctival sac of each guinea pig's right eye, and the left eye was injected with NaCl as a control (*n* = 5 mice in each group). Guinea pigs were observed at 24, 48, and 72 h after inoculation for signs of infection and inflammation in their eyes and assigned scores as follows: “–” for normal eye indistinguishable from the contralateral uninoculated eye, “+” for lacrimation or eyelid edema, “++” for lacrimation or eyelid edema plus mild conjunctival hyperemia, “+++” for lacrimation or eyelid edema with mild conjunctival hyperemia plus slight exudates, and “++++” for full-blown purulent keratoconjunctivitis.

### Mouse infection

Six-week-old Balb/c female mice weighing ~20 g obtained from the Animal Center of the Academy of Military Medical Sciences were anesthetized by diethyl ether. A bacterial suspension of 20 μL was applied intranasally to each mouse with a pipette. A group of eight mice were challenged with 10^6^ CFUs. Lungs were collected from all animals 24 h after infection, washed with PBS to remove contaminating blood, and homogenized. The wild-type and Δ*Ssr1* samples were serially diluted and the resulting colonies were counted on Brain Heart Infusion plates with and without kanamycin. Results were recorded by the competition index. For survival studies, the wild-type and Δ*Ssr1* were introduced intranasally at 10^6^ CFUs. Ten mice per group were used in these studies. Mice were monitored daily for survival. The statistical analysis was performed using the log-rank (Mantel-Cox) test.

### sRNA targets prediction

The sTarPicker prediction method was used to predict the target mRNAs of *ssr1* against the entire genome of the wild-type (Vandal et al., 2009). This genome-wide prediction application is available at http://ccb.bmi.ac.cn/starpicker/prediction.php. The program is based on a two-step model of hybridization between an sRNA and a target. In comparisons with different sRNA target prediction tools, such as IntaRNA, TargetRNA, and sRNATarget, we found that sTarPicker performed best in both the accuracy of predicted binding sites and in identification of sRNA targets on an independent test dataset (Ying et al., 2011).

A 2-DE analysis was performed according to previously described methods with slight modifications (Li et al., 2011; Zhou et al., 2011). In brief, the prepared pooled protein samples (600 mg protein on preparative gels or 120 mg protein on analytical gels) were mixed with rehydration buffer to a volume of 450 mL. The IPG strips (pH 4–7, 24 cm, GE Healthcare, 17-6002-46) for the first dimension were used to isolate the altered proteins, and the running condition was set at 20°C, step 1: 300 V for 0.5 h, step 2: 700 V for 0.5 h, step 3: 1500 V for 1.5 h, step 4: 9900 V for 3 h, step 5: 9900 V for 6.5 h, step 6: 600 V for 20 h, and step 7: 8000 V constant for a total of 56,000 Vh. After completion of the isoelectric focusing program, the strips were equilibrated in two steps: 15 min in an immobilized pH gradient equilibration buffer [6 M urea, 2% SDS, 30% glycerol, 0.375 M Tris (pH 8.8), 20 mg/mL DTT, and a trace of bromophenol blue] and then alkylated for 15 min. Subsequently, a 12.5% SDS-PAGE 2-DE was performed. Electrophoresis was carried out at 20 mA per gel for 40 min and then at 30 mA per gel until the dye front reached the bottom. The protein spots were visualized via either silver staining or Coomassie Brilliant Blue G-250 staining. Triplicate 2-DE gels were performed for each group.

Triplicate gels from Δ*Ssr1* and wild type strain (control) were analyzed for spot intensity using Image Master 2D Platinum software (GE Healthcare, 28-9408-30) according to the protocols provided by the manufacturer. The criterion for significant changes in protein expression was a difference of at least 1.5-fold between the Δ*Ssr1* and wild type strain groups.

### Protein identification

The protein identification was performed according to the method described by Shi et al. (2009). Gel spots showing significant changes were excised from 2-DE gels. Gel spots were washed and then digested with sequencing-grade trypsin. MALDI-TOF MS and TOF/TOF tandem MS were performed on a MALDI-TOF-TOF mass spectrometer (4800 Proteomics Analyzer, Applied Biosystems,). The instrument was set in reflector mode. Peptide mass fingerprints coupled with peptide fragmentation patterns were used to identify the protein in the International Protein Index (IPI) database using the MASCOT search engine. The functions of the identified proteins and their associated biological processes were matched by searching Gene Ontology (http://www.geneontology.org).

### Ethics statement

The animals were obtained from the laboratory animal center (Academy of Military Medical Sciences). The methods were carried out in accordance with the approved guideline of Academy of Military Medical Sciences. The experimental protocol was approved by the Ethics Committee for Animal Experimentation of the Academy of Military Medical Sciences.

## Results

### Novel small regulatory RNA (sRNA) in *S. flexneri*

**Figure 1:**
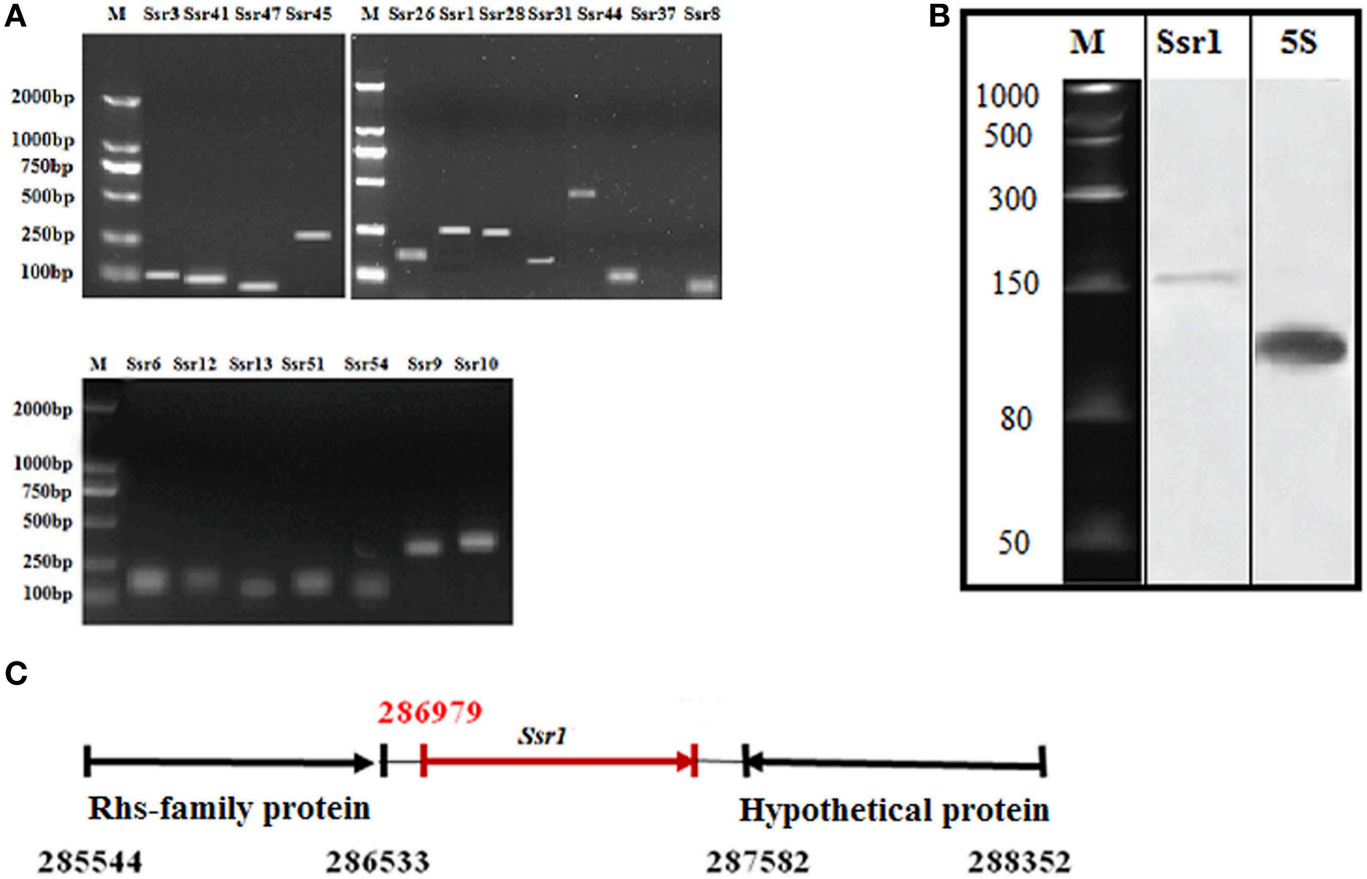
**Experimental verification and expression of *Shigella flexneri* sRNAs. (A)** RT-PCR of 22 sRNAs expressed in the wild-type grown to the late stationary phase; M denotes molecular weight marker (DL2000, Takara); **(B)** Northern blot of a novel sRNA (*Ssr1*) in *S. flexneri*. *Ssr1* was verified by northern blot in the wild-type grown to the stationary phase; M denotes RNA molecular weight marker; 5s RNA was used as control; **(C)** *Ssr1* positional information in the *S. flexneri* genome by 5′RACE.

Using a transcription unit-based method, we predicted 57 sRNAs in the intergenic regions of the *S. flexneri* 2a 301 strain (the wild-type) (NC_004337). These predicted sRNAs are referred to hereafter as “*Ssrs*” (for *Shigella* small RNAs). The sRNAs varied from 50 to 500 nt in length and they were numbered from 1 to 57 (Table 1). To test whether all these regions express detectable transcripts, RT-PCR was performed, and 18 sRNAs were then validated (Figure 1A). According to its high level of expression in *S. flexneri*, we opted to perform northern blot for *Ssr1*, which revealed that the length of small RNA (*Ssr1*) was ~150 bp (Figure 1B). We conducted homology analysis in other enteric bacteria, and found that *Ssr1* only exists in *Shigella.*

**Table 1:** **Small RNAs (sRNAs) in *Shigella flexneri* predicted in this study**.

**sRNAgenes**	**Adjacent genes**	**Strand**	**5′end**	**3′end**	**RT-PCR**
*Ssr1*	SF0268/yafV	→ → ←	286,849	2,87,502	Yes
*Ssr2*	SF0490/SF0491	← → ←	508,677	5,08,786	
*Ssr3*	ybfA/kdpA	← → →	624,431	6,24,666	Yes
*Ssr4*	nagD/asnB	→ → →	652,524	6,52,614	
*Ssr5*	dacA/ybeD	→ → →	687,772	6,87,859	
*Ssr6*	SF4458/SF0680	→ → ←	712,385	7,12,787	Yes
*Ssr7*	ipaH_2/ybhE	← → →	921,042	9,21,488	
*Ssr8*	SF0948/rlmL	← → →	994,696	9,94,779	Yes
*Ssr9*	ymbA/fabA	→ → ←	1,002,529	1,002,752	Yes
*Ssr10*	icdA/SF1156	→ → →	1,197,645	1,198,294	Yes
*Ssr11*	SF1205/ychF	← → ←	1,252,826	1,253,554	
*Ssr12*	pfkB/SF1508	← → →	1,540,588	1,540,702	Yes
*Ssr13*	ydeJ/SF1559	← → →	1,591,049	1,591,209	Yes
*Ssr14*	ribE/ydhE	← → →	1,723,061	1,723,156	
*Ssr15*	SF1779/tehB	← → ←	1,817,490	1,817,786	
*Ssr16*	SF1897/SF4470	→ → ←	1,936,312	1,936,490	
*Ssr17*	SF2011/insA	← → →	2,036,426	2,036,668	
*Ssr18*	yejH/rplY	→ → →	2,305,600	2,305,712	
*Ssr19*	SF2423/vacJ	→ → ←	2,474,415	2,474,548	
*Ssr20*	ddg/SF2445	→ → ←	2,499,782	2,500,097	
*Ssr21*	sseA/sseB	→ → ←	2,638,950	2,639,193	
*Ssr22*	SF2969/SF2970	→ → ←	3,065,046	3,065,180	
*Ssr23*	sap/SF2991	→ → →	3,087,241	3,087,450	
*Ssr24*	SF3002/SF3003	→ → →	3,099,131	3,099,293	
*Ssr25*	ygjR/ygjT	→ → →	3,228,567	3,228,805	
*Ssr26*	SF3873/hemY	← → ←	3,994,443	3,994,974	Yes
*Ssr27*	engB/yihI	← → →	4,063,618	4,063,972	
*Ssr28*	yjdA/phnA	← → →	4,281,467	4,281,794	Yes
*Ssr29*	SF4216/SF4217	→ → →	4,390,809	4,390,927	
*Ssr30*	dcuA/aspA	← → ←	4,470,403	4,470,496	
*Ssr31*	pagP/dcuC	←← →	6,94,274	6,93,980	Yes
*Ssr32*	SF4458/SF0680	→ ←←	7,12,535	7,12,712	
*Ssr33*	mdoH/SF1046	→ ←←	1,090,831	1,091,008	
*Ssr34*	SF1350/insB	→ ←←	1,397,476	1,398,217	
*Ssr35*	SF1542/ycgW	→ ← →	1,576,030	1,576,603	
*Ssr36*	ycgW/SF4467	→ ←←	1,577,114	1,577,901	
*Ssr37*	ydeJ/SF1559	←← →	1,591,802	1,591,911	Yes
*Ssr38*	ydbK/SF1823	→ ← →	1,860,960	1,861,117	
*Ssr39*	SF1879/ipaH_4	→ ← →	1,917,499	1,917,778	
*Ssr40*	SF1927/SF1928	→ ← →	1,963,576	1,963,685	
*Ssr41*	yecI/SF1950	→ ← →	1,984,123	1,984,327	Yes
*Ssr42*	nmpC/SF1978	→ ←←	2,010,014	2,010,161	
*Ssr43*	SF2038/SF4477	← ← ←	2,060,687	2,060,776	
*Ssr44*	SF2042/SF2043	← ← ←	2,063,884	2,064,349	Yes
*Ssr45*	fadI/fadL	←← →	2,469,554	2,469,983	Yes
*Ssr46*	SF2493/SF2494	→ ← →	2,554,471	2,554,606	
*Ssr47*	sseA/sseB	→ ←←	2,638,501	2,638,972	Yes
*Ssr48*	rpsP/ffh	← ← ←	2,744,850	2,744,947	
*Ssr49*	stpA/SF2698	←← →	2,772,656	2,772,940	
*Ssr50*	insA/yghK	← ← ←	3,107,699	3,106,244	
*Ssr51*	SF3060/sufI	← ← ←	3,156,062	3,156,664	Yes
*Ssr52*	greA/dacB	←← →	3,320,988	3,321,087	
*Ssr53*	yhhX/yhhY	←← →	3,553,565	3,553,647	
*Ssr54*	hdeD/yhiE	→ ← →	3,635,036	3,635,153	Yes
*Ssr55*	yhiX/yhiW	→ ← →	3,698,133	3,698,286	
*Ssr56*	shiD/insB	→ ←←	3,817,761	3,817,881	
*Ssr57*	yijP/ppc	← ← ←	4,165,277	4,165,400	

We determined the transcriptional start site of *Ssr1* by using rapid amplification of 5′ cDNA ends (5′RACE) analysis. 5′RACE analysis identified the *Ssr1* 5′ end, which is located at 286979 (Figure 1C). *Ssr1* is located in an intergenic region between two open reading frames, SF0268 (encoding a putative Rhs-family protein) and SF0269 (encoding a hypothetical protein). To investigate the role of *Ssr1* in *S. flexneri*, a mutant, Δ*Ssr1*, was derived from the wild-type by deleting the *Ssr1* sequence and replacing it with a kanamycin resistance sequence. Additionally, an *Ssr1* complementation strain was constructed by transforming the plasmid pACYC184 containing the *Ssr1* gene into the *Ssr1* mutant strain to assess the complementation of function in trans.

### *Ssr1* is a novel factor involved in the response to acidic stress

**Figure 2:**
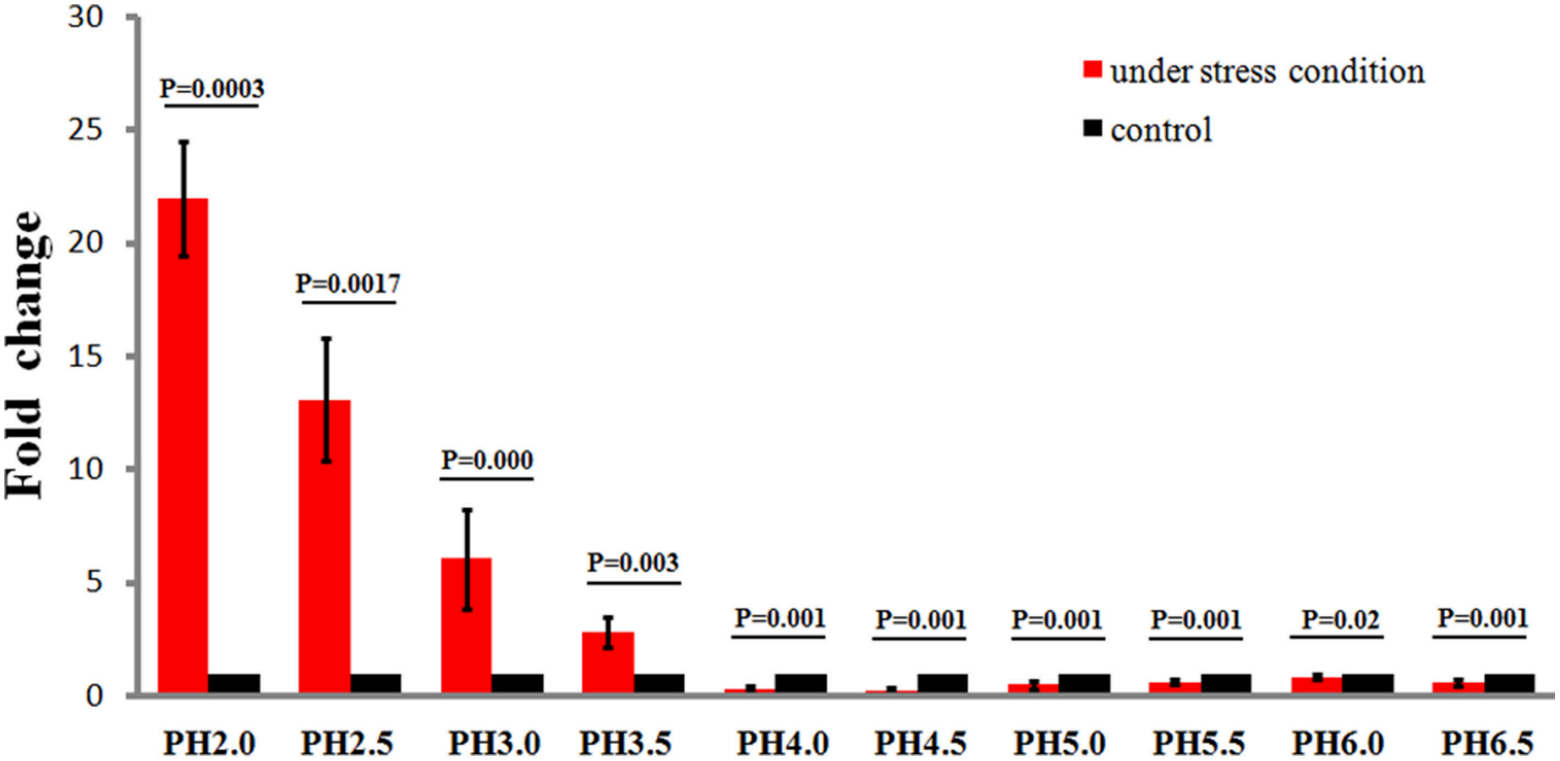
**Relative transcription of the sRNA *Ssr1* in the wild-type when exposed to a range of pH conditions for 30 min**. The wild type was cultured in LB broth buffered to a range of pH values. Expression of *Ssr1*: red bars indicate fold changes calculated as means from triplicate experiments, representing the ratios of *Ssr1* expression levels under various pH conditions compared with pH 7.0. Standard deviations are indicated by the error bars. Black bars represent the control transcript values. The statistical analysis was performed using the Student's *t*-test.

To study the effects of different acidic stress conditions on *Ssr1*, we exposed *S. flexneri* to media of different pH. qRT-PCR revealed that *Ssr1* was most highly expressed in the pH 2.0–4.0 range. The highest expression was at pH 2.0 compared with the expression level at pH 7.0, and expression significantly declined from pH 4.5 to 6.5 (Figure 2). Interestingly, a strong linear correlation between pH value and *Ssr1* expression was observed (*R* = 0.785, *P* < 0.05). These results suggest that *Ssr1* may be highly expressed in the stomach of the host (~pH 1.5–3.5) during an *S. flexneri* infection. Thus, *Ssr1* expression may be regarded as a response to an acidic environmental change as the bacterium colonizes the gastrointestinal tract.

### Reduced stress tolerance and survival of the *S. flexneri Ssr1* mutant

**Figure 3:**
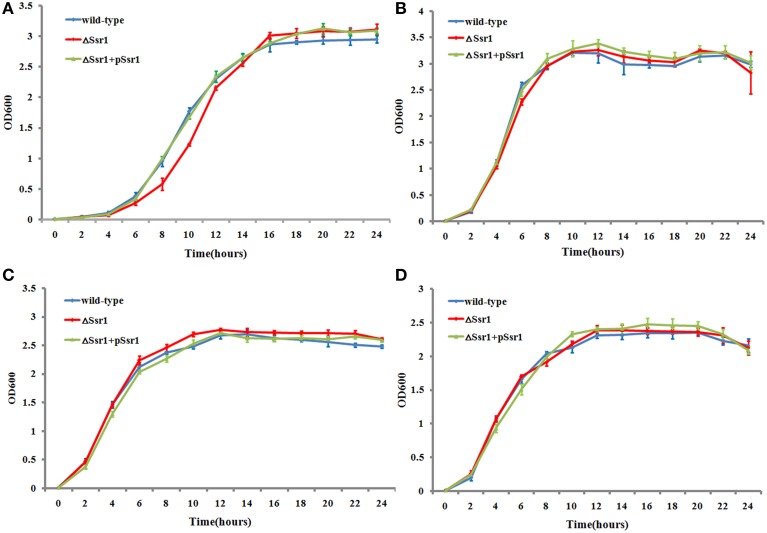
**Acidic stress tolerance of *Shigella flexneri***. Growth characteristics of the wild-type, Δ*Ssr1*, and complementation strains in LB under different pH levels: **(A)** pH 5.0; **(B)** pH 6.0; **(C)** pH 7.0; and **(D)** pH 8.0. The error bars indicate standard deviations based on duplicate experiments.

The ability of enteric bacteria to thrive in the extremely acidic environment of the stomach is crucial for colonization and survival in the intestine (Hoe et al., 2013). To investigate these functions of *Ssr1*, we successfully created Δ*Ssr1* and a plasmid-based complementation strain of *Ssr1*. The effects of *Ssr1* on *S. flexneri* growth in acidic media were then examined. Δ*Ssr1* grown in medium adjusted to pH 5.0 exhibited a significantly prolonged lag in the exponential phase compared with the wild-type (*P* < 0.05) (Figure 3A). Under pH 6.0, 7.0, and 8.0, Δ*Ssr1* entered exponential phase at 2 h and entered a stationary phase at 10 h, with the wild-type exhibiting the same growth tendency (Figures 3B–D). There was no significant difference between the growth of the wild-type and the *Ssr1* complementation strains under the above conditions.

**Figure 4:**
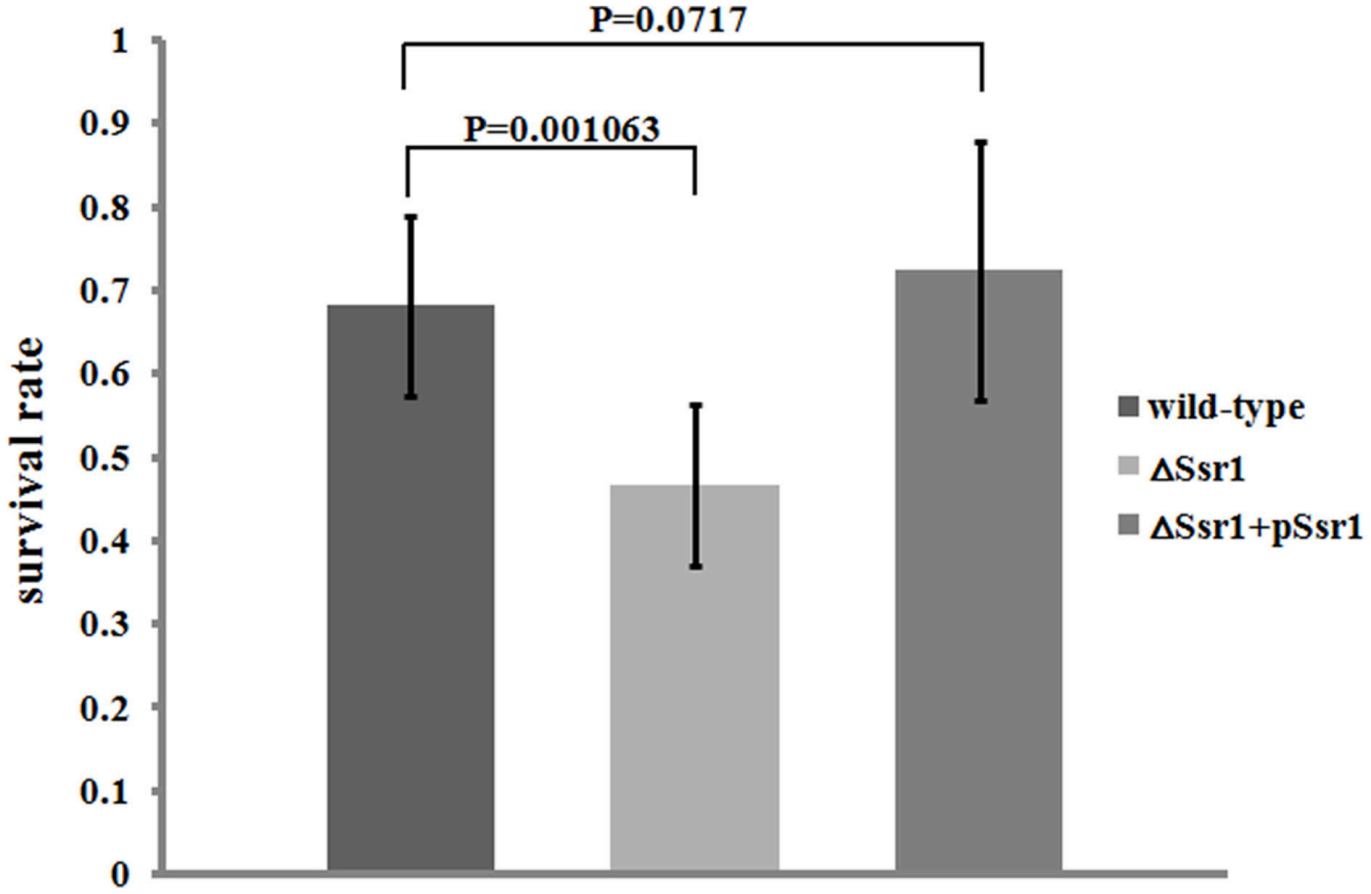
**Survival of Δ*Ssr1* relative to the wild-type when exposed to acid stresses**. Wild-type, Δ*Ssr1*, and complementation (Δ*Ssr*1+ p*Ssr1*) strains were grown in LB (pH 7.0) to the exponential phase and then subjected to acidic stress (pH 3.0) conditions. Recovered colony-forming units were determined by counting plated serial dilutions as described in the Materials and Methods. Bars represent the mean percent survival compared with untreated controls. Each assay was conducted in three replicates. A statistical analysis was performed using the Student's *t*-test.

*S. flexneri* can survive various stresses, including that engendered by low pH (Teixeira-Gomes et al., 2000). To further determine the role of *Ssr1* in the acidic stress tolerance of *S. flexneri*, the survival rates of Δ*Ssr1, Ssr1* complementation, and the wild-type during acidic stress (pH 3.0) were compared. This revealed that the survival rate of Δ*Ssr1* decreased by 22% under low pH stress (*P* < 0.05), and the complementation strain exhibited no significant difference (Figure 4). This result indicated that *Ssr1* in *S. flexneri* plays an important role in the resistance to acidic stress.

### The novel *Ssr1* regulates virulence in *S. flexneri*

**Figure 5:**
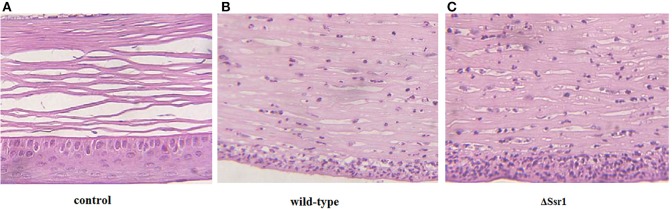
**The corneal pathology of the *Shigella flexneri* wild-type and Δ*Ssr1***. Inflammatory cells are more in Δ*Ssr1* than in the wild-type strain, while the NaCl control group has no inflammatory cells. **(A)** In the blank group **(B)** the wild-type group **(C)** the mutant Δ*Ssr1* strain.

To determine whether *Ssr1* is important for virulence, guinea pig conjunctival sacs were injected with Δ*Ssr1* and the wild-type. Guinea pigs inoculated with the wild-type developed slight conjunctivitis without purulence at 24 h post-infection, which developed to keratoconjunctivitis with purulence after 48 h, and continued to be severe at 72 h. Guinea pigs inoculated with Δ*Ssr1* displayed a severe keratoconjunctivitis with purulence at 24 h, and at 48 and 72 h, the situation was increasingly severe. The NaCl control group did not develop conjunctive inflammation (Table 2). Then, the eyes of the experimental guinea pigs were analyzed by biopsy, and hematoxylin and eosin staining. The eyes of guinea pigs infected with both the wild-type and mutant showed an inflammatory reaction such as corneal epithelial cell necrosis, shedding, and corneal intrinsic membrane fiber disorder, accompanied by bleeding and inflammatory cell infiltration. But the inflammation of the guinea pigs carrying Δ*Ssr1* was more serious than that of infected with the wild-type (Figure 5). The NaCl control group elicited no inflammatory response in this test. Together, these results suggest that *Ssr1* expression may be related to the virulence of *S. flexneri*.

**Table 2:** **Keratoconjunctivits in guinea pigs inoculated with wild-type and Δ*Ssr1*, as well as a NaCl control**.


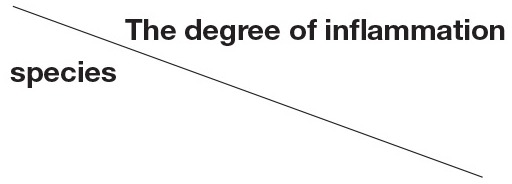	**Time**														
	**24 h**					**48 h**					**72 h**				
	**1**	**2**	**3**	**4**	**5**	**1**	**2**	**3**	**4**	**5**	**1**	**2**	**3**	**4**	**5**
Control	—	—	—	—	—	—	—	—	—	—	—	—	—	—	—
Wild-Type	+	+	+	+	+	++	++	++	++	++	+++	+++	+++	++++	++++
Δ*Ssr1*	++	++	++	++	++	+++	+++	+++	+++	+++	++++	++++	++++	++++	++++

A sereny test shows the virulence of sf 301 and Δ Ssr1 strains. Mouse keratoconjunctivitis was rated as follows: “-” for normal eye indistinguishable from the contralateral uninoculated eye, “+” for lacrimation or eyelid edema, “++” for lacrimation or eyelid edema plus mild conjunctival hyperemia, “+++” for lacrimation or eyelid edema with mild conjunctival hyperemia plus slight exudates, and “++++” for full-blown purulent keratoconjunctivitis.

**Figure 6:**
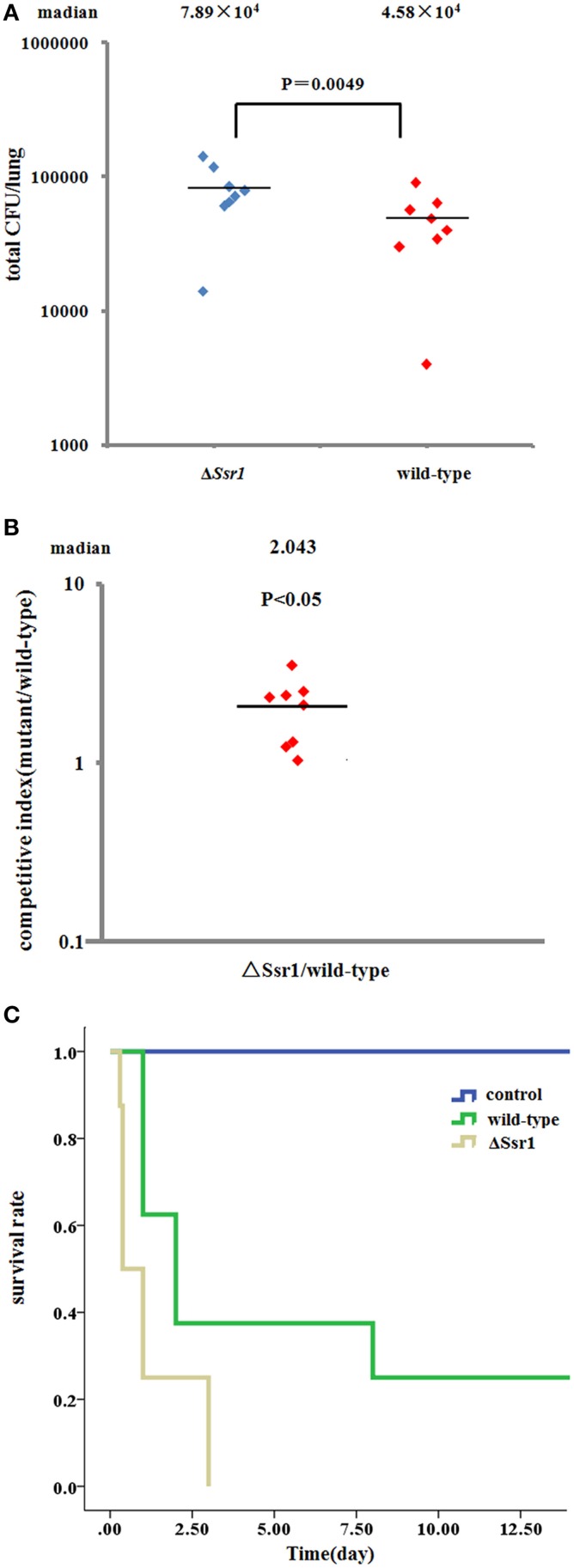
**Mice were infected with the *Shigella flexneri* Δ*Ssr1* and wild-type strains**. Bacterial density was measured 24 h post-infection on Brain Heart Infusion (BHI) and BHI with kanamycin plates. **(A)** The total colony-forming units in the lungs of mice infected with the Δ*Ssr1* and wild-type strains (two sample *t*-test, *P* = 0.0049); **(B)** A competitive index of 1 represents equivalent amounts of wild-type and Δ*Ssr1*. The competitive index of the wild-type and Δ*Ssr1* is greater than 1 (*P* < 0.05); and **(C)** the survival rates of the Δ*Ssr1* and wild-type strains.

To further confirm the keratoconjunctivitis results, the wild-type and Δ*Ssr1* were tested in a mouse lung invasion assay. The number of total Δ*Ssr1* colony-forming units (CFUs) recovered after gentamicin treatment to a mean value of 7.89 × 10^4^. This was significantly higher (*P* = 0.0049) than the number of the wild-type CFUs (4.58 × 10^4^) (Figure 6A). The calculated confidence interval median value was 2.043. This strongly suggests that Δ*Ssr1* has an impact on the capability of *S. flexneri* to effectively invade cultured cells (Figure 6B).

To further determine the pathogenic role of *Ssr1 in vivo*, BALB/c mice were infected intranasally with the wild-type and Δ*Ssr1*, and survival was monitored. Survival rates of 62.5% (5/8 mice) infected with the wild-type and 25% (2/8 mice) infected with Δ*Ssr1* were observed at 1 day post-infection (log-rank test, *P* = 0.00). At 3 days post-infection, the group infected with Δ*Ssr1* had a mortality rate of 100% (8/8), while the mice infected with the wild-type strain had a mortality rate of 62.5% (5/8) (Figure 6C).

### Identification of *Ssr1* targets

**Figure 7:**
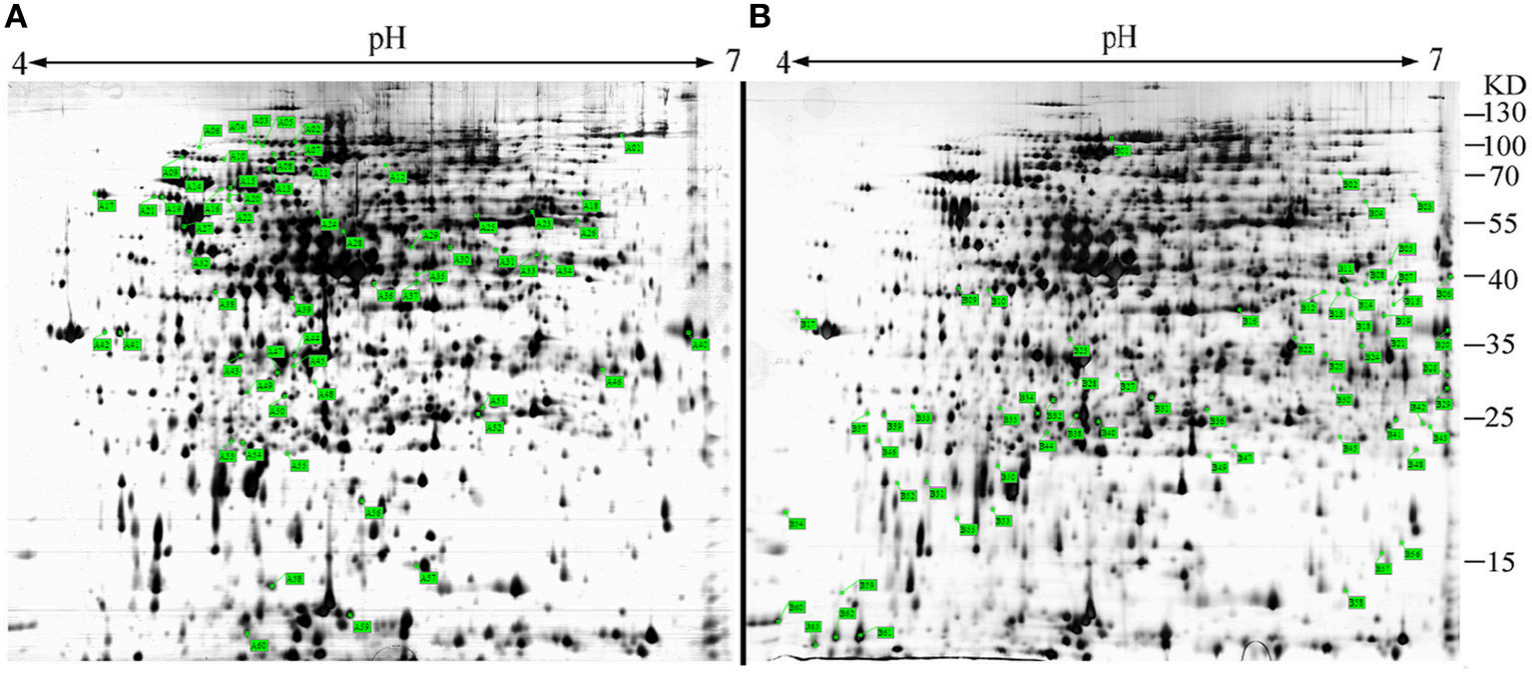
**Representative two-dimensional gel electrophoresis of the wild-type and Δ*Ssr1***. The two figures represent differential protein expression levels in the wild-type **(A)** and Δ*Ssr1* **(B)** in the stationary phase. The highlighted spots in **(A)** represent proteins that have greater expression levels in the wild-type, whereas those in **(B)** represent proteins that are more abundant in the Δ*Ssr1*.

sRNAs usually regulate other genes at a post-transcriptional level by directly or indirectly interacting with the associated mRNA. To identify the targets of *Ssr1* sRNA, we used sTarPicker to search the regions of *S. flexneri* mRNAs for potential RNA duplex formation with *Ssr1.* This analysis predicted 283 binding sites and suggested that *Ssr1* sRNAs possible interact directly with these target mRNAs via a common region. Subsequently, the two-dimensional gel electrophoresis (2-DE) technique was used to compare Δ*Ssr1* to the wild-type to confirm these targets. For the proteomic analysis, total proteins were harvested during the stationary phase of bacteria. Protein spots with a change of more than 1.5-fold were selected for further analysis. The 2-DE maps of the wild-type and Δ*Ssr1* are shown in Figure 7. Detailed information on the up- and downregulated proteins is listed in Table 3.

**Table 3:** **Differentially expressed proteins in Δ*Ssr1*—*Shigella flexneri***.

**Spot no**.	**NCBI GI identifier**	**Protein description**	**Average ratio**	**pI**	**Mr**	**Protein score**	**Final_Localization**
**DOWN-REGULATED PROTEIN SPOTS IN Δ *Ssr1* MUTANT**							
A04	gi|56479706	Leucyl-tRNA synthetase	1.92316	5.11	97,815	154	Cytoplasmic
A07	gi|24112341	Aminopeptidase	1,000,000	5.12	99,418	250	Cytoplasmic
A09	gi|24111612	Outer membrane protein assembly factor YaeT	1,000,000	4.93	90,611	318	OuterMembrane
A10	gi|24112901	Phosphoenolpyruvate synthase	3.50291	4.93	87,809	222	Cytoplasmic
A14	gi|24111463	Molecular chaperone DnaK^*^	1.92652	4.83	69,142	201	Cytoplasmic
A19	gi|24113761	Phosphoenolpyruvate-protein phosphotransferase	1.94119	4.78	63,722	172	Cytoplasmic
A25	gi|56480123	GTP-binding protein Der	1,000,000	5.52	55,089	295	Cytoplasmic
A26	gi|24115037	ATP synthase F0F1 subunit alpha	2.13811	5.8	55,416	140	Cytoplasmic
A29	gi|24115456	Peptidase PmbA	1.88864	5.48	48,624	220	Cytoplasmic
A36	gi|24111974	Galactokinase	1.70124	5.36	41,928	384	Cytoplasmic
A37	gi|24113879	Bifunctional nitric oxide dioxygenase/dihydropteridine reductase 2	2.42852	5.49	43,998	94	Cytoplasmic
A40	gi|56479896	Glyceraldehyde-3-phosphate dehydrogenase (GAPDH-A)	2.00224	6.61	35,681	67	Cytoplasmic
A41	gi|24112338	Outer membrane protein F	1.84728	4.76	39,339	316	OuterMembrane
A42	gi|24113600	Porin	2.37934	4.56	41,377	249	OuterMembrane
A43	gi|56479781	Outer membrane protein OmpA^*^	1.79543	5.65	37,374	273	OuterMembrane
A47	gi|24114191	Agmatinase	3.24396	5.14	33,764	112	Cytoplasmic
A48	gi|56479821	DNAse	1,000,000	5.13	30,233	122	Cytoplasmic
A49	gi|24115117	Phospholipase A	1,000,000	5.15	33,142	184	OuterMembrane
A50	gi|24113681	Histidine ABC transporter substrate-binding protein HisJ	1,000,000	6.77	29,067	176	Periplasmic
A51	gi|56480449	Uridine phosphorylase	1.73923	5.81	27,341	114	Cytoplasmic
A53	gi|24112822	Hypothetical protein SF1441	2.04377	5.5	27,813	92	OuterMembrane
A55	gi|24114860	Glutathione S-transferase	1,000,000	5.10	22,762	220	Cytoplasmic
A56	gi|24113642	Hypothetical protein SF2345	1.83302	5.29	11,280	240	Unknown
A58	gi|24114909	Deoxyuridine 5′-triphosphate nucleotidohydrolase (dUTPase)	1.97105	5.05	16,433	381	Cytoplasmic
A59	gi|24115554	30S ribosomal protein S6	2.41987	5.26	15,177	81	Cytoplasmic
A60	gi|24112560	Cell division topological specificity factor MinE	2.01343	5.15	10,286	57	Cytoplasmic
**UP-REGULATED PROTEIN SPOTS IN Δ *Ssr1* MUTANT**							
B02	gi|31983589	Hypothetical protein CP0125	7.76534	6.13	70,080	161	Extracellular
B03	gi|24112624	Nitrate reductase 1 subunit beta	8.3085	6.36	59,012	437	CytoplasmicMembrane
B04	gi|24114695	Glycogen synthase	2.9026	6.05	52,919	143	Cytoplasmic
B05	gi|24113822	Hypothetical protein SF2538	2.71421	7.08	53,874	147	Unknown
B06	gi|24114874	L-lactate dehydrogenase	1,000,000	6.33	42,902	303	Cytoplasmic
B09	gi|31983568	Mxi-Spa secretion machinery protein	4.36788	4.82	40,280	196	Extracellular
B10	gi|24114573	DNA-directed RNA polymerase subunit alpha (RNAP subunit alpha)	2.32256	4.98	36,717	417	Cytoplasmic
B16	gi|31983586	Hypothetical protein CP0126	3.34543	5.65	36,696	169	Extracellular
B19	gi|24111977	Phospho-2-dehydro-3-deoxyheptonate aldolase	2.61593	6.14	38,399	115	Cytoplasmic
B20	gi|56479896	Glyceraldehyde-3-phosphate dehydrogenase	2.05812	6.61	35,681	315	Cytoplasmic
B22	gi|24113759	Cysteine synthase A	1,000,000	5.83	34,553	300	Cytoplasmic
B24	gi|24112168	Glycosyl transferase	2.81361	6.08	35,303	78	Cytoplasmic
B26	gi|24114611	FKBP-type peptidylprolyl isomerase	3.85906	8.39	28,910	206	Cytoplasmic
B29	gi|56479617	30S ribosomal protein S2	2.10231	6.61	26,812	232	Cytoplasmic
B37	gi|24114648	Phosphoglycolate phosphatase	1,000,000	4.58	27,414	192	Cytoplasmic
B40	gi|56480611	Purine nucleoside phosphorylase	6.93801	5.42	26,147	383	Cytoplasmic
B41	gi|24112000	Succinate dehydrogenase iron-sulfur subunit	1.96182	6.32	27,393	336	CytoplasmicMembrane
B43	gi|24112177	Glutamine ABC transporter ATP-binding protein	1,000,000	6.25	26,699	90	CytoplasmicMembrane
B48	gi|24115367	LexA repressor	6.58657	6.23	22,344	472	Cytoplasmic
B52	gi|24114332	Esterase	6.63036	4.61	21,742	99	Cytoplasmic
B54	gi|24115223	Ribonuclease activity regulator protein RraA	2.76987	4.07	17,464	109	Cytoplasmic
B57	gi|56479823	Hypothetical protein SF1112 (UPF0227 protein YcfP)	2.99923	6.13	21,441	302	Cytoplasmic
B58	gi|24113666	Hypothetical protein SF2370(UPF0304 protein YfbU)	2.19077	5.95	19,649	255	Cytoplasmic
B61	gi|31983576	IpgC, cytoplasmic chaperone for IpaB and IpaC	5.56608	4.58	17,916	343	Cytoplasmic

* *Represent the potential targets of Ssr1, which were predicted by sTarPicker method*.

**Figure 8:**
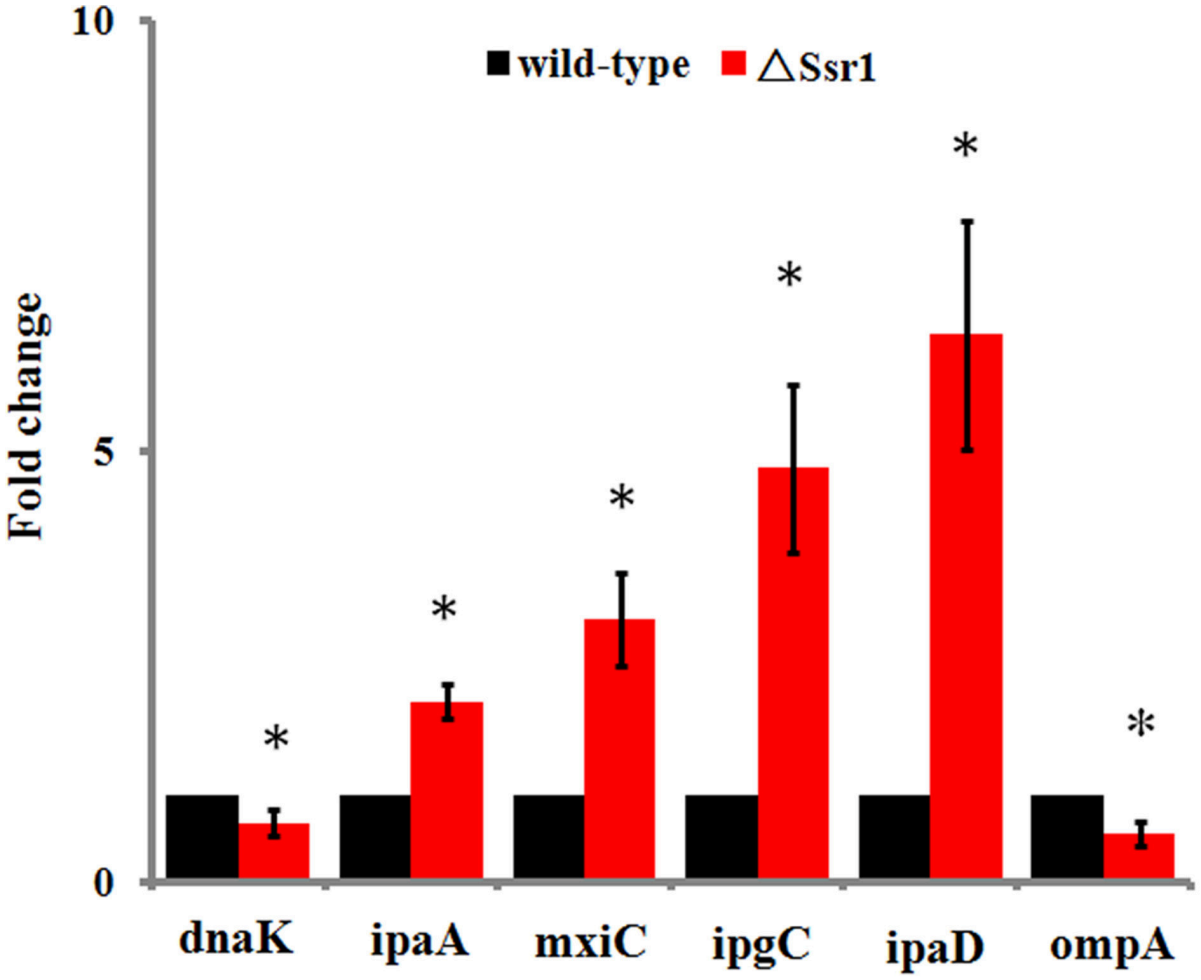
**qRT-PCR for six mRNAs that are targets of *Ssr1***. Standard deviation is indicated by the error bars. Statistical analysis was performed using the Student's *t*-test. Data are representative of three independent experiments. ^*^Represents statistical significance.

Fifty-one differentially expressed proteins were successfully identified. Among them 24 proteins were upregulated and 27 proteins were downregulated in Δ*Ssr1* (Table 3). The identified proteins were mainly involved in amino acid transport and metabolism, cell wall, carbohydrate transport and metabolism, and energy production. The proteins had diverse cellular locations, including the cytoplasm and cell membranes, while others were secreted, and participate in various metabolic pathways that are regulated by sRNAs, suggesting that *Ssr1* has a role in the modulation of bacterial colonization and pathogenicity. The level of a stress response protein (DnaK) was increased 1.92-fold compared with Δ*Ssr1*. DnaK is upregulated in order to protect cells from several stress conditions (Tomoyasu et al., 2012). Another important protein is the OmpA, which was increased 1.80-fold compared with Δ*Ssr1*. The downregulated proteins included those involved in the T3SS response compared with Δ*Ssr1*. IpaA, ipaD, ipgC, and mxiC were downregulated 7.76, 3.39, 5.56, and 4.36-fold, respectively. These T3SS factors are indispensable for virulence in *S. flexneri*. Using qRT-PCR, we analyzed the expression of six genes at pH = 7.0 (outer membrane protein OmpA, the general stress response molecular chaperone Dnak, and IpaA, IpaD, IpgC, and MxiC; these last four genes belong to the T3SS and are thus important in virulence). The transcriptional levels of these genes in the mutant were calculated relative to those in the wild type. Accordingly, the levels of these genes in the wild type were set to 1 as reference (Figure 8). The results were consistent with those of the 2-DE analysis, suggesting that *Ssr1* modulation of stress resistance and virulence occurs in part by regulation of these proteins. By integrating the results of the sTarPicker and 2-DE analysis, 14 mRNAs (*pheT, pepN, ppsA, dnaK, ptsI, engA, galK, ompA, ycfH, yibF, dut, minE, rpsB*, and *ycfP*) may be direct targets of *Ssr1*. Our results provide evidence that *Ssr1* in *S. flexneri* is involved in multiple physiological and biochemical processes, and has a particularly important role in stress response and virulence processes, via modulation of the above targets.

## Discussion

The attenuation of acidic stress is a key component of *Shigella* responses that determine its pathogenicity, since the bacterium must face the environmental conditions of the stomach prior to infecting the colon. In turn, activation of acidic stress resistance provides cross-protection against other environmental stresses, such as oxidation, osmotic pressure, and heat stress (Foster and Spector, 1995; Vandal et al., 2009). sRNAs are involved in the toleration of environmental stresses, and thereby contribute to the virulence of several pathogens (Marteyn et al., 2012). Here, we demonstrate that the sRNA Ssr1 play critical roles in responding to acidic stress tolerance and virulence in *S. flexneri*. To our knowledge, this study provides the first functional bioinformatics and wet-lab analysis of a novel sRNA (*Ssr1*) in *S. flexneri*. *Ssr1* was highly expressed across a range of pH values, and the growth and survival of Δ*Ssr1* was reduced under acidic stress conditions when compared with the wild-type strain. *Ssr1* is strongly upregulated during acidic stress, which leads to increased tolerance; this comes at the expense of reducing the virulence and pathogenicity of *Shigella*.

To adapt and survive in a complex hostile environment, bacteria have to adjust their gene expression levels through regulatory networks, and this process affects the host infection process, which is particularly important for pathogenesis (Papenfort and Vogel, 2010). To fully understand the function of *Ssr1* in both the stress response and virulence, the regulatory pathway of regulators of *Ssr1* must be identified. Here, we found that DnaK, a member of the heat shock protein 70 (HSP70) family that assists in the refolding and hydrolysis of abnormal proteins (Zhang et al., 2014), was upregulated by *Ssr1* based on the results of qRT-PCR and proteomic analysis. The expression of HSP70 family members is increased following exposure to stress, including that induced by pH changes (Tomoyasu et al., 2012). Remarkably, in *S. flexneri*, acidic stress induced robust expression of *Ssr1*, which then may directly upregulated DnaK protein; this reveals a mechanism by which *S. flexneri* produces a stress response protein in order to survive in acidic conditions. The proteomics results revealed that *Ssr1* negatively regulates the *ipa* and *mxi* genes of the T3SS system, which is required for the invasion of the colorectal epithelium and for promoting virulence in *S. flexneri*. However, these T3SS-related genes were predicted as indirect targets of *Ssr1* by sTarPicker, implying that the precise virulence mechanism mediated by *Ssr1* requires further clarification. We suggest that regulation of *E. coli* OmpA, an outer membrane protein that mediates a wide-range of activities including resistance to complement, and invasion and survival within host cell, is a candidate mechanism. OmpA can regulate T3SS to influence virulence in *Yersinia* (Bartra et al., 2012). In addition, ompA expression is increased by *Ssr1* in *S. flexneri*, and we demonstrated that this contributes to the negative regulation of T3SS genes. Thus, we conclude that *Ssr1* regulation enables a rapid response to various environmental conditions, yet can also decrease the expression of T3SS in *S. flexneri*. And, once the bacteria enter normal conditions, *Ssr1* becomes refractory to downregulation, which leads to increased T3SS activation. The identities of all the molecular components of pathways used by bacteria to improve their tolerance while reducing their virulence under extreme environments are still unclear.

Our study of *Ssr1* provides new insights into the interactions between enteric bacteria and the host environment. Although we did not validate all of the genes we identified as targets that participate in the regulatory network, our results indicate that *Ssr1* sRNAs have roles in regulating genes involved in virulence and stress tolerance, specifically in response to acidic stress. These findings will help us better understand how this bacterium responds to and regulates pathogenicity under diverse environmental stress conditions. Our report also enhances the understanding of the virulence mechanisms employed by *S. flexneri*, and reaffirms the concept that bacteria use multiple strategies to modulate their pathogenesis in order to survive and thrive. However, additional evidence is needed to confirm the relationship between the stress response, stress tolerance, and virulence. *In vivo* experiments that address how the role of Ssr1 affects virulence during acid stress will be particularly important. We have planned further functional and mechanistic studies of sRNA regulatory networks in *S. flexneri*, in order to determine how their interactions affect *S. flexneri* infections. Through such studies, novel approaches to treat and control *S. flexneri* outbreaks, such as by targeting acidic storage, may be identified. Finally, we note that our bioinformatics sRNA prediction method can also be used to identify virulence regulators in other bacteria, and may thus be a powerful tool for this research community.

## Author contributions

HS, LW, and WL designed the research, assessed and interpreted the results, and prepared the manuscript. LW, GY, LQ, SQ, PL, RH, LJ, ZW, and XD carried out the data analysis and designed experiments. GY, XL, JX, and LW assisted in the experiments.

### Conflict of interest statement

The authors declare that the research was conducted in the absence of any commercial or financial relationships that could be construed as a potential conflict of interest.
