# Supplementary material for: A Novel Small RNA Regulates Tolerance and Virulence in Shigella flexneri by Responding to Acidic Environmental Changes
Source: Front Cell Infect Microbiol. 2016 Mar 8;6:24. doi: 10.3389/fcimb.2016.00024 (PMC4782007; doi:10.3389/fcimb.2016.00024)
Supplement: Supplementary file 1 [file Table1.DOCX]

Table S1. All Primer in this study

| Primer | | | sequence (5’-3’) | | | Primer | | sequence (5’-3’) | | | |  |  |  |
| --- | --- | --- | --- | --- | --- | --- | --- | --- | --- | --- | --- | --- | --- | --- |
| **RT-PCR** | | | | | | | | | | | |  |  |  |
| Ssr1-F | TGCGGAAAAAAATGGACAAT | | | | | | Ssr1-R | | | AGTTAAGGGGATGCGAGACG | | | | |
| Ssr2-F | AAAAAAAGCAACAGCGTA | | | | | | Ssr2-R | | | ACAGGAGTCGCTGAAAAA | | | | |
| Ssr3-F | TACTTTTTTTACACCCCG | | | | | | Ssr3-R | | | CTATCACGCCTGCACTCA | | | | |
| Ssr4-F | CACCAATCGACAATCACC | | | | | | Ssr4-R | | | TAAAAAAATGCCGCAAAG | | | | |
| Ssr5-F | GTGTAATTTCCGTCCCCATA | | | | | | Ssr5-R | | | ATAGCAACTCCCGCCAGA | | | | |
| Ssr6-F | ATAATGAAATGCCCCCTC | | | | | | Ssr6-R | | | GAAAAAACACGCACGACT | | | | |
| Ssr7-F | TTCTCCCCCCCGACTTTTGA | | | | | | Ssr7-R | | | TCCCCGCTTTTGTTTACCTG | | | | |
| Ssr8-F | AACTTTATGACATGACGCG | | | | | | Ssr8-R | | | AATAGCCTAACGGGACAG | | | | |
| Ssr9-F | TTTGTTCCTCTTCACATTTT | | | | | | Ssr9-R | | | GAATCATAACTGCCGTCAAT | | | | |
| Ssr10-F | CCAGCCATCCGTCATCCATA | | | | | | Ssr10-R | | | CTTACACCGCCTCTCAGCCC | | | | |
| Ssr11-F | TGGTTATCATTTTCATCTTGCC | | | | | | Ssr11-R | | | CCACTACACATCAGCCCTCTTC | | | | |
| Ssr12-F | TACATTGCTAAGTTATTCGC | | | | | | Ssr12-R | | | ACAACAAAAAAAGAGACCAT | | | | |
| Ssr13-F | TTGCTTTTTCTGTATGCTCG | | | | | | Ssr13-R | | | AATCGGATTTATATGTTTGA | | | | |
| Ssr14-F | ATGGGCAGCAAAAAGAAGAA | | | | | | Ssr14-R | | | ATGGGCAGCAAAAAGAAGAA | | | | |
| Ssr15-F | AGGGGCTAAGTCAATCATCA | | | | | | Ssr15-R | | | TTACTGTTCTTCTTTTCGGT | | | | |
| Ssr16-F | CAGCAATCTGAGTAGCGAAA | | | | | | Ssr16-R | | | AGGACCGTAGAAAAAAGGAC | | | | |
| Ssr17-F | GATTATGTTCTCGCTCATGGAC | | | | | | Ssr17-R | | | ACTCAGGCTGGTGTTATGTTTT | | | | |
| Ssr18-F | GAGTATAGAATCTCGCCCGC | | | | | | Ssr18-R | | | CGACCCAGCAACAGGTAAGT | | | | |
| Ssr19-F | TTATCTCCTTTTACTTGTGG | | | | | | Ssr19-R | | | TTTTTTGTTGTTATCCCTCA | | | | |
| Ssr20-F | AATGCCTCTTGTCTCGTTTC | | | | | | Ssr20-R | | | TTCACTTTACATCTCTCCGC | | | | |
| Ssr21-F | TTGTCAAGCAAAAGAGAGTT | | | | | | Ssr21-R | | | AGTACGATACAGAAGCACCG | | | | |
| Ssr22-F | CGTGTTGTAATGTTCATAAGCG | | | | | | Ssr22-R | | | CAACAAAATAAATCAACCGT | | | | |
| Ssr23-F | TATGCTAATAATAACTCCTG | | | | | | Ssr23-R | | | TCTGTTGTTCCCTGAATCTC | | | | |
| Ssr24-F | TATCAAAAGGGACTGCGGGTAT | | | | | | Ssr24-R | | | GGTTCTTCTAAATAGTGGTGCC | | | | |
| Ssr25-F | AGGGGAGTAACTTCATTGCC | | | | | | Ssr25-R | | | TGTGGTTTTTCACTCATCGT | | | | |
| Ssr26-F | CGTTTTCAATCCTACCTCTG | | | | | | Ssr26-R | | | TGTAATCCTCTTTGCTTCCT | | | | |
| Ssr27-F | CGGTTGATTGTTTGTTTA | | | | | | Ssr27-R | | | ATCCTCTGTCTTCGCCTC | | | | |
| Ssr28-F | CGTCTTCTGATGCGTTTA | | | | | | Ssr28-R | | | AATGCTCTACTTGTTCTT | | | | |
| Ssr29-F | CTTGTTGTAGAAGCCGTTAT | | | | | | Ssr29-R | | | AAGATTCGTCCGGTTGTT | | | | |
| Ssr30-F | TTACTGCTCACAAGAAAA | | | | | | Ssr30-R | | | AAAAGGCACGTCAGATGA | | | | |
| Ssr31-F | AGACCCAACATAAAGAATAA | | | | | | Ssr31-R | | | ATCTCGTAAAAAAAATAAAA | | | | |
| Ssr32-F | AGGACCGTAGAAAAAAGGAC | | | | | | Ssr32-R | | | CAGCAATCTGAGTAGCGAAA | | | | |
| Ssr33-F | GGCTCTTTTTCGGGGCTGTG | | | | | | Ssr33-R | | | ATCCGTTTTGTGGTGTTTTT | | | | |
| Ssr34-F | GACGGCACTGGTAACGAGGA | | | | | | Ssr34-R | | | TGAGAGGTGGCGAGGGATTT | | | | |
| Ssr35-F | ATACCTTCCATTTGACTG | | | | | | Ssr35-R | | | GGTTTTGTGTTTTTTCTG | | | | |
| Ssr36-F | TACCTACTCGCTGATTGCCC | | | | | | Ssr36-R | | | GTGGTGGTTGTTGCTTTCCC | | | | |
| Ssr37-F | AAAAAAAACAACAGCGTA | | | | | | Ssr37-R | | TATTTTTCAGCGACTCCT | | | | | |
| Ssr38-F | CGGGCTTGTCTTTTTATA | | | | | | Ssr38-R | | CATTGCCACTGATTTTCC | | | | | |
| Ssr39-F | GAAGACAAAAAGATACGCAA | | | | | | Ssr39-R | | TATGAAAAAAGGAGCTGAAG | | | | | |
| Ssr40-F | TATTTTGCACAGGAGTCGCTGA | | | | | | Ssr40-R | | GTTGTCGCCATTGTTCGATTAA | | | | | |
| Ssr41-F | ATACTTTTCAGGATTTTGCG | | | | | | Ssr41-R | | TGATTTTTGTGATGGTTGTC | | | | | |
| Ssr42-F | ACTATCTTGCTTCTGACTAT | | | | | | Ssr42-R | | TTGTTCACTGAGTATTATTA | | | | | |
| Ssr43-F | ACTGTGAGTGGTTTGTTGGATA | | | | | | Ssr43-R | | AAAGCGGGCGAAACGATA | | | | | |
| Ssr44-F | AAAACAAAACCCGCCGAAGC | | | | | | Ssr44-R | | GCAGATAGAGAAAAGCCCCA | | | | | |
| Ssr45-F | GCGTTTGTTCTGCTTCTG | | | | | | Ssr45-R | | GTTTATCTGGCACCTTGG | | | | | |
| Ssr46-F | GAAGCGGGAAATAGAGAC | | | | | | Ssr46-R | | AAGGTAATAGCGGGATAA | | | | | |
| Ssr47-F | ATTGTTTATTTAGCGTAT | | | | | | Ssr47-R | | AGAATTTTTAATTTTGAG | | | | | |
| Ssr48-F | AAAACAACCGGACCCCATCG | | | | | | Ssr48-R | | TCTCCAGAAATCAGTAAAAT | | | | | |
| Ssr49-F | TCACTGGTGCTTTCTGCT | | | | | | Ssr49-R | | CTAATGAAAGTTTGGGGGGGAG | | | | | |
| Ssr50-F | TCAACAGACACAAACAGGCA | | | | | | Ssr50-R | | CTCCAACAACACACCAATCA | | | | | |
| Ssr51-F | ATTATCTGGCGTGTAGGC | | | | | | Ssr51-R | | CGAATCTGTTTTTTGGTG | | | | | |
| Ssr52-F | GCAAAAAAATACCGACCC | | | | | | Ssr52-R | | AGTAAACGATGACCCTTC | | | | | |
| Ssr53-F | AAAAAAAGCCAGCACCCG | | | | | | Ssr53-R | | GAACCTGAAAGCACGACA | | | | | |
| Ssr54-F | TTTAAATCGGATTACATTTT | | | | | | Ssr54-R | | CAGTTTGTGATCTCTGAAGA | | | | | |
| Ssr55-F | CTCCAGACTAATAAACCG | | | | | | Ssr55-R | | AACTTACTGAGAGCACAA | | | | | |
| Ssr56-F | AAAAACGCCAGCAACACA | | | | | | Ssr56-R | | ATTGACAACGCTAACTAT | | | | | |
| Ssr57-F | GTCTTGAAAAAAATATCGCC | | | | | | Ssr57-R | | CTTCTGTTCTAACACCCTCG | | | | | |
| **Northern blot probe** sequence (5’-3’) | | | | | |  | | |  | | |  |  |  |
| Ssr1-p | | GTCATTACCACGAACTTCCCACATTG | | | | | | | | |  |  |  |  |
| 5S-p | | GTTTCACTTCTGAGTTCGGCATGGGGTCAGGTGGG | | | | | | | | |  |  |  |  |
| **RACE** sequence (5’-3’) | | | | | | | | | | | |  |  |  |
| Ssr1-5’RACE outer primer | | | | | CTCCAGTCATTACCACGAAC | | | | | | | |  |  |
| Ssr1-5’RACE inner primer | | | | | TACCACGAACTTCCCACATTGC | | | | | | | |  |  |
| **qRT-PCR** | | | | | sequence (5’-3’) | | | | | | | |  |  |
| Ssr1-qRT-F | | | | | TGCGGAAAAAAATGGACAAT | | | | | | | |  |  |
| Ssr1-qRT-R | | | | AGTTAAGGGGATGCGAGACG | | | | | | | |  |  |  |
| 16s-F | | | | | CAGCCACACTGGAACTGAGA | | | | | | | | |  |
| 16s-R | | | | | GTTAGCCGGTGCTTCTTCTG | | | | | | | | |  |
